# Methodological Bias That Can Reduce (or Affect) the Process of Diagnostic Construction in Clinical Settings

**DOI:** 10.3389/fpsyg.2019.00157

**Published:** 2019-02-05

**Authors:** Antonio Iudici, Elena Faccio, Gianluca Castelnuovo, Gian Piero Turchi

**Affiliations:** ^1^Department of Philosophy, Sociology, Education and Applied Psychology, University of Padova, Padova, Italy; ^2^Psychology Research Laboratory, Istituto Auxologico Italiano (IRCCS), Milan, Italy

**Keywords:** bias (B), diagnosis, evaluation, clinical, assessment

## Diagnosis as a Process

The diagnostic process is a fundamental element of practice in the clinical and healthcare fields in particular, both for its repercussions on patient healing and care and for the professionalism with which healthcare professionals carry out their roles. Deriving from the Greek *dia-gnosis* (knowing through), the diagnostic process is the cognitive process implemented by a healthcare professional in his or her work. Although “diagnosis,” at the level of common sense, is more widely used as a noun, it should be noted that the diagnostic process recalls the path of discovery implemented by professionals in reference to their categories of observation, leading to a definition of their work object. As part of an operation on the cardiovascular system, a surgeon will mainly work on the biochemical implications of that body part, while a physician struggling with the decision to conduct a plastic surgery on the face could have as an object not only the face but also the story of the person requesting the intervention. On closer inspection, the physician's observational and professional skills interact with the patient's communicative skills, and this exchange may occur according to different interactive modalities (compliance). Indeed, information on some medical history could only emerge with by creating the favorable relational conditions (Kripalani et al., 2007; Sanders, 2009; Castelnuovo, 2013). Furthermore, this interactive dynamism is influenced by the context in which it occurs and by the objectives of the healthcare structure. Far from being a mechanistic action, the diagnostic process entails a broad and varied complexity, which requires a suitable management capacity (decision making). Research in the healthcare field has found that 44% of the general errors made by healthcare personnel concern the diagnostic process (Shiff et al., 2009). One-third of these errors, as some studies claim, has a cognitive nature (Pepe et al., 2012), and the greatest difficulties concern the processing of the contents found (Turchi and Perno, 2004; Croskerry, 2008). This demonstrates the need to increasingly refine the reasoning at the base of the diagnostic process.

In this work, we intend to highlight the possible critical implications during the gnoseological process performed by the staff in clinical and healthcare contexts and, at the same time, to systematize some methodological principles useful to reduce the so-called *Cognitive Dispositions to Respond* (CDRs), which can lead to diagnostic errors.

## What Triggers CDRs in the Diagnostic Process

In the cognitive pathway that characterizes the diagnostic evaluation, we can, therefore, find some typical cognitive errors generated by different factors, which include: previous experience, interaction with the patient, delegation to the instruments used, bias, confusing the symptoms with the disease, and so on. These errors, since they are not always perceived, risk generating improper choices and activating expensive and irrelevant practices, such as inviting to new investigations and using new instruments (Hall et al., 2015). For instance, the selective attention toward specific observation criteria and the attitude of those who try to recognize the prototype of the disease in the narration of the other can lead to the risk of not grasping anything else and determining so-called cognitive illusions (Most et al., 2005). This implies the risk of reasoning through expectations (*ascertainment bias*), leading to further investigations in order to confirm the expectation rather than to disconfirm the data through further tests (Crupi et al., 2006).

## Losing Sight of the Fact That the Patient's Contribution is Determined by the Interaction That is Created

In many cases, the effectiveness of the psychological and medical intervention is influenced by the patient's contribution, both in the acquisition of anamnestic elements and the way in which the patient interacts with the physician or the psychologist (*unpacking principle*^1^). For instance, the place hosting the interview can or cannot facilitate a climate of privacy (Vermeire et al., 2001), can be hospitable and lead to greater confidentiality (FitzGerald and Hurst, 2017), or can involve a certain inclination to hurriedness (Ha and Longnecker, 2010). It has also been found that verbal and non-verbal communication can affect the interaction, facilitating or not facilitating narrations, descriptions, and explanations (Kripalani et al., 2007). In addition, the physician, while acquiring information and content, simultaneously defines the interactive structure. For instance, closed questions in the form of interrogation can put the patient in a passive position, with the consequence of limiting his or her responsibility in managing his or her own health (Sanders, 2009). Conversely, open questions that can involve the patient in his or her report can lead to greater patient participation, an indicator of which is his or her inclination to ask questions (Doyle et al., 2013). Some researches highlight a number of critical aspects in interrupting the patient's narration (Dyche, 2005), hence the importance of acquiring the data that the person considers useful, according to the way and timing with which he or she considers fit to communicate them. One implication of all this is that the clinician can “de-inspire” himself with regard to data acquisition by considering the patient uncooperative or by focusing on the content alone and not on the process, for instance. One way to manage this implication might be to ask the patient for feedback on their the relationship with the clinician—whether they are comfortable, or whether there are aspects inhibiting them from expressing themselves freely.

## Delegating one's Assessment and Choices to the Instrumentation Used (test, etc.)

Although technology, instruments and tests have enriched the list of useful tools that help in conducting a precise diagnosis, it is equally useful to remember how sometimes operators confuse their instruments with their decisions. This delegation involves some cognitive distortions and, in general, a surrender of responsibility. This critical aspect is substantiated by the idea that the test or the instrumentation are based on specific assumptions that need to be mastered in order to avoid reifying data that is not neutral, but exclusively defined by the investigated construct. If we think of a personality test and its results, regardless of the theoretical definition that establishes the personality construct, we risk objectifying a result that is not objective at all. Various authors consider replacing the evaluation of the clinician with test evaluations or instrumental examinations a serious mistake (Pepe et al., 2012). It could be said that, in extreme cases, a physician or a psychologist without a test is better than a testing operator without medicine or psychology. The negative implications concern the indiscriminate use of text tools and the idea that the data can be considered absolute and objective. One way to overcome these implications may be to constantly bring the test result back to the assumptions it is based on and, if used at the same time, to relate the assumptions of the tests used (and not only the results) in order to provide a more coherent and precise meaning to the results.

## Confusing the Disease or the Symptoms With the Patient

In many cases, the idea of having to arrive at an understanding of the problem experienced by the patient leads the healthcare professional to make the patient correspond to the symptoms described through the prototype of the hypothesized disease. A story based on the absence of friends, on the absence of external experiences, the feeling of social isolation, can give rise to the conviction of having a depressed person (*representativeness restraint*). The diagnostic prototype guides the exploration of the anamnestic history, determining, in fact, the confusion between what emerges in the narration and what we want the narration to produce. It is a process of self-referential research rather than of an acquisition based on doubt (*commission bias*). This often leads to confusing the disease with the patient (*fundamental attribution error*). However, it is widely demonstrated that there is no correlation between the patient's clinical status and the degree of the disease or between the report of a problem and the disorder (Larson et al., 1996). This error is sometimes triggered by the nosographic process, i.e., to relate to the problem presented by the patient based on diagnostic prototypes from the manuals.

The negative implications involve the possibility for the professional relate to the ùpatient based on the symptoms identified or the diagnostic category attributed without grasping the meaning that the individual (patient) attributes to those symptoms or the category nor how he uses symptoms or category (*diagnosis momentum*). One way to address these implications may be to pay attention to the manner in which the patient is described in their context and to bring out biographical elements within which some actions implemented by the person may find meaning. It might also be useful to discuss the interaction between the individual and the people significant for him/her, since aspects that can be configured in relational terms and not only in personological terms may emerge.

## Choosing Based on one's Experience and not one's Expertise

Some studies (Grooopam, 2008) highlight the tendency of healthcare professionals to make decisions based on their own experience. Experience is certainly an important aspect for a professional, but it can lead to some cognitive distortions, linked to an assessment based on what we know more than a critical evaluation. Often this aspect is intertwined with another error, namely that of excessively estimating one's own abilities (*overconfidence bias*). As a matter of fact, the overestimation of some evaluations is considered one of the main causes of error (Fischhoff, 1988). Paradoxically, this aspect, regarding those who have the most experience, leads to the conviction of becoming more knowledgeable and to expect a reoccurrence of the events experienced in their personal professional activity (Motterlini and Crupi, 2005).

The critical implications of this are to assess on the basis of personal experience and the cases dealt with, which are limited and partial. To overcome this implication, it may be useful to engage with colleagues or a team on a regular basis and use “third-party” or evidence-based elements valid for the area in question (Iudici et al., 2015).

## Exasperated Research of the ETIOLOGY and Causes

The need for productivity and a sub-specialist culture, together with other social factors, have probably contributed to the frantic search for causes by the clinician with respect to the patient. If, in the medical field, this aspect is more justified, in the psychological field it is extremely critical, considering that many believe there are no psychological disorders with a definite etiology (Turchi and Della Torre, 2007; Iudici, 2015). What we want to highlight here is the importance of not considering etiology in ideological terms (Pepe et al., 2012), because the essential factor in the gnoseological process is correct data, even if this does not make it possible to complete the gnoseological framework (*premature closure*). Indeed, considering as certain something that is actually incomplete can reduce the possibility of significant investigations, while an admittedly incomplete framework can leave space for the evolution of the cognitive pathway (Jena et al., 2011). Diagnosis is always a hypothesis and the clinician must know how to simultaneously manage this climate of uncertainty, flexibility and intrinsic evolutionary possibility of the diagnostic process. The negative implication is to speed up the diagnostic process rather than to arrive at a comprehensive framework of the data and to believe that without understanding the cause, a decision cannot be taken or uncertainty cannot be managed. One possible way to overcome this problem is to use the elements present and focus on data acquisition rather than the outcomes.

## Bias TOWARD the Patient

Although a professional can consider himself or herself prepared to avoid bias, no one can really be considered free from the risk of assessing people in a distorted manner. This is because, among the various types of bias that a healthcare worker can have, there are some, defined implicit (FitzGerald and Hurst, 2017), characterized by the way we define specific narrative links between events. For example, some authors have found that a cause of error concerns the embarrassment that is found in visiting certain types of patients based on gender (Bönte et al., 2008; Jost et al., 2009), age (Nosek and Riskind, 2012), profession (Nosek et al., 2007), weight (Sabin et al., 2012), and hygienic conditions (Holroyd and Sweetman, 2016) and even on how much the patient's name is familiar based on the geographic origin (Rooth, 2010).

From these implications, a distorted interpretation of the problem presented, based on personal associations and prejudices, may emerge (Martin et al., 2014). One way to address this problem would be to recognize the prejudices we are most exposed to. The recognition of the prejudices can happen if you are willing to believe that categorization is a useful yet a not always precise cognitive process.

## Conclusions

This work does not claim to systematize all the possible types of bias in the diagnostic process, but focuses on the main distortion phenomena which, since they cannot be eliminated, must at least be taken into account in daily clinical work.

The importance of this communication, therefore, concerns the need to raise awareness among healthcare professionals regarding monitoring the different types of bias that can be activated while carrying out their work, its implications and to identify potential solutions (Stone and Moskowitz, 2011). A broad and well-established sector literature (Zestcott et al., 2016) emphasizes that, although professionals consciously try not to succumb to errors, distortions and bias, no one can really consider himself or herself immune from them, considering that the cultural process in which we are socialized reorganizes groups in a stereotypical way, intervening primarily in the implicit aspects of our categorisations (Romaioli et al., 2008; Salvini et al., 2012).

It is for this reason that several scholars have formalized some specific anti-bias training to reduce the potential effect of social stereotypes (Dasgupta and Greenwald, 2001; Devine, 2001; Rudman et al., 2001; Dasgupta, 2009; Joy-Gaba and Nosek, 2010; Lai et al., 2013; Gonzalez et al., 2014; Byrne and Tanesini, 2015).

Ten years after the publication of Kahn's masterful paper (2008) on the importance of an etiquette-based rather than evidence-based clinical-psychological medicine, it is necessary that critical work such as this make clinicians reflect on ordinary procedures that are too often encaged in guidelines, protocols, and manuals without a real contact with the patient. When we assimilate clinical practice and diagnosis, we implicitly accept a vision of the individual based on the deficit as a deviation from normality. On the other hand, taking into account the specificity, uniqueness, and complexity of the patient means adopting a diagnostic and therapeutic process tailored to the needs of the person and not on the pathology within which the person is often framed (Carli, 2008; Faccio et al., 2016a,b; Iudici and Gagliardo Corsi, 2017; Iudici et al., in press). Paraphrasing the words of Alessandro Salvini, an Italian clinical epistemologist, a diagnostic process limited to reassuring and shared nosographic categories without considering the patient's narration makes the clinician “gain certainty but lose intelligence” during his or her daily work.

## Author Contributions

AI has been involved in the Study Conception and Design. EF, GT, and GC have been involved in the analysis and interpretation of data and the drafting of manuscripts. EF, GT, GC, and AI have been critical of the critical revision.

### Conflict of Interest Statement

The authors declare that the research was conducted in the absence of any commercial or financial relationships that could be construed as a potential conflict of interest.

## Footnotes

^1^For a list of the main clinical biases, see Graber et al. (2002).
